# The Beautiful Death

**Published:** 1867-10

**Authors:** 


### THE BEAUTIFUL DEATH.

ON entering her teens, she was thrown into the society of men, young and old, of wealth, wit and position, but whether young or old, all had either read TCM PAINE at home, or in pursuing their professional education in Paris, after the Revolution, had imbibed the atheistical ideas of the times, and their banterings of the good, their really stale jests of the clergy, of the Sabbath-day and of Divine Revelation,—so impressed her young mind that, although afterwards she was brought under the influence of the powerful teachings of such men as TYNG the elder, to whose communion she attached herself, she never, through a life of nearly thirty years succeeding, was able at all times, to shake from her mind the doubts which had found lodgement in her heart so long before. She never ceased to lament and mourn over those doubts, as obstacles to that greedy and pleasurable drinking in of Bible truths, which is the inestimable blessing of those who, from the first lispings of the alphabet and the Lord's Prayer at a mother's knee, have learned to feel that there is no higher authority for man than that "The Bible says so."

### LIFE A PUPILAGE.

One day I was explaining to her that there was a fixed design on the part of God in reference to his children, to order all the incidents of life in such a manner as to cause them to make it more certain that they should attain future happiness at last; and that although they might not be able to see what possible influence or bearing some of life's experiences could have in such a direction, yet the assurance had been given by the Divine Teacher, "What I do thou knowest not now, but shall know hereafter," and perhaps one of the sources of pleasure in the future state would be in looking back on life's history and tracing on a map, as it were, how it was that the incidents of our pilgrimage all converged to the great central point of making salvation sure.

"I wish I could look upon these things as you do," and it was spoken so mournfully as to almost force the exclamation, "Thrice blessed are they who are early trained to an implicit confidence in all that the Bible says and are thoroughly kept from "evil associations" at an impressible age.

### IS DEATH AN AGONY?

A few days before she died she looked me full in the face and said, "If it is not wrong to say it, but I fear it is, I would rather go." The incubus of her life had been "the dying agony;" the act of death had been associated in her mind with heavings and writhings and gaspings for breath, amounting to intolerable torture. The philosophy of a true physiology had often been brought to bear on her understanding while in health in favor of the position that when struggles and even convulsive contortions were witnessed in the last hour, they occurred at so late a period that the system had lost its susceptibility to feeling, precisely as in an epileptic fit, where the subject seems to be writhing in agony for some minutes, and yet, on the instant of regaining consciousness, there is not the slightest remembrance of anything that occurred during the interval, and this seems to be proven by the imperturbable calmness which seems to imbue the whole being, bodily and mental, immediately succeeding the attack. But when that important event came to her and it was evident that the soul was on the wing, just hovering over the confines of time, she said to me, "Is dying so easy as this?" And then, in allusion to the seemingly cherished apprehensions of her life, she added, "How could I expect that." Raising her hands and looking at her finger nails, she said, "How far up have the limbs become cold?" Thus the mind seemed in the act of dissolution to be as clear as a bell, in contemplating the physiological phenomena of the system.

### THE CLAIMS OF SCIENCE.

The disease had been of such a character that for several months the best medical and surgical minds in the nation were unwilling to express themselves definitely as to its exact nature; the general conclusion, however, was that a fatal result was inevitable in four or five months; and with certain complications, very much sooner.

Not long after that, in conversation about its possible nature, she said, "I would like to know what it is; and if it will promote the cause of science, you may have an examination after death, especially as it may throw some light upon any ailment that may befall the children hereafter. I would as lief be cut up as not — there can be no feeling in the dead body—but be sure that the arteries are divided." This she said in reference to a long indulged fear that she might be buried alive. After this, she gave directions to her eldest daughter where she would find a particular piece of linen, which should be placed under her body during dissection, to prevent any blood dropping upon the carpet. That a frail woman, with all the instinctive timidity peculiar to her sex, should have the moral heroism on the very last day of her life, to discourse in this manner on such a subject, indicates a vigor of intellect, and a clearness of mind, rising above the ruins of a wasted body, which could belong only to one "whose heart is fixed, trusting in the Lord."

### THE ANGEL'S WING.

Shortly before she died she said to her sister, interrupting a train of conversation upon a wholly different subject, "There, Mary! I see that wing again,—the very tip of it—over my right shoulder. I wonder what it can be? It seems to be the wing of a bird just flapping, as if it were about to alight; or like a white banner fluttering in the wind: it seems as white as snow; but whenever I turn my head to get a better sight of it, it disappears." This sister, who had no patience with those who believed in anything "supernatural," replied, "I guess it is nothing. I see nothing, and it is bright sunshine; but if anything could attract dear mother to this earth again, it would be a scene like this, one sister from a distance, sitting by the other, who was so ill."

Sometime before that, a favorite cousin who had come a long distance to see her had, without her knowledge, taken a lock of her hair to a lady "Medium," saying to her only this: "I have a cousin who is ill, and wish to know thy opinion of her condition." Pressing it to her forehead for a moment she replied, "I seem to see leaves falling. If I had seen a coffin or a spade, I should conclude she would die very soon; as it is, I infer she may live until the Autumn, when the leaves begin to fall." The cousin and the 'Medium' did not meet again for some weeks, and then, in a distant city, on a ferry boat; when the 'Medium' said, as if continuing the former conversation, "I saw your daughter (recently dead,) this morning; she is dressed in white, and decked with flowers; she seems busy, and says she is preparing to receive an elderly person, and I should think it was your cousin." Four hours before, that cousin had died: neither of them could have known the fact.

There are many persons in high health, and in full exercise of whatever intelligence they may possess, who could weave this narration into a very plausible recital. But when the invalid was made acquainted with the first part of the statement, she seemed grieved, and, withal, a little offended, and dismissed it curtly by saying, "I wish to have nothing to do with such things; they look too much like having communication with the Evil One;' and it was never alluded to thereafter, except once, when she said, "I shall not live until the fall of the leaf," nor did she.

READ ME THE PROMISES.

As showing how little disposition she had to look to, or lean on anything not immediately connected with the Bible, a very beautiful article, written by one of our most eminent clergymen, on the designs of affliction, was read to her ; she did not wait to hear it out, and with a little impatience said, "These are mere opinions ; read me the promises ; get the Bible ; these are but the conjectures of men."

About twenty-four hours before she died, the family, supposing the end was at hand, assembled around the bed ; she gathered in a moment what was passing in their minds, and seemed surprised at their thinking she was near her end, and said, "I was only in a state of suspension, but I don't thank him for bringing me back," alluding to what the physician had done, with that object in view.

GOING ABROAD.

In early life she had been sent abroad in one of her uncle's ships, and although "fete"-ed on his account partly, but more from the vivacity of her character, the unusual cultivation of her mind, and her extraordinary conversational power, aided by the personal attraction of a natural ruddiness of cheek, in contrast with her long, luxuriant black hair, and teeth that vied in whiteness with polished ivory, and as solid, continuing to the last day of life, tall and attractive in form and figure, she was not, with all these. carried away beyond herself; nor did she abandon herself to the blighting, corrupting and dissipating influence of 'Society.' On the contrary, she took time to keep a diary throughout her journey, besides writing numerous letters to friends at home, some of which the writer has seen for the first time, within a week, and which are certainly more entertaining and instructive, although written thirty-nine years ago, than most of contributions of "Foreign Correspondents" of the present time. On her return home she attached herself to the communion of the church, there to abide for life.

THE BLISS OF DYING.

The film of death, like the shadows of the grave, were evidently gathering over her eyes, for although it was broad day, she thought night was closing in, and the body seemed to be in that delightful state which all have experienced in the act of falling asleep after a fatiguing day, and she would say to her husband as his head laid upon her pillow, from time to time, in a cheery, trusting tone, after any little conversation, "Come, now, let us go to sleep." Thus, instead of the last agonies of expiring nature, which had been so vividly portrayed to her imagination during life, she had vouchsafed to her the "bliss of dying." After this, both body and mind fell into a state of greater quiet and repose, from which she gradually passed into the act of death, without apparent consciousness, and by shorter and shorter breaths, as gentle, seemingly, as the sleeping babes', until finally, without the shadow of a struggle in foot or finger or limb; without a gasp or groan, or even a grimace, the last breath came, and on Saturday, July 27, 1867, at five in the morning, she literally "fell asleep," and doubtless into the arms of attending angels, sent to Pilot her, as was done to her ascending Master, through realms of space, to the mansions of the blessed, to "no more go out."

### THE MACHINE OF LIFE.

A frequent desire of her life was that her sickness might be a protracted one, not only as a means of ensuring an easy death, as she expressed it, "by the machine wearing out gradually in all its parts, thus enabling it to cease its running without shock or jar," but as affording her a full opportunity and abundant leisure to make the necessary preperation of heart and soul for the untried future. She had had her desire. In her six month's illness, she had not, except in the first few days, any special, pain; there was a persistent burning sensation, and occasional and darting twinges—nothing more—but a continous feeling of distention and discomfort. Thus was she doubly blessed, in being able in perfect calmness, first to dispose of her estate, and then to give her mind wholly to the more important concerns of an endless existence.

### DOUBTS AND FOREBODINGS.

In consequence, seemingly, of the want of an early religious education, and the unfortunate early associations alluded to, this lady's whole religious life was clouded with doubts and despondency; she never had any lively, self-supporting, instinctive faith: she wanted to believe; was always striving to believe, and had an abiding distress that she had not "a firmer faith in God," his providence and his grace; and her aim was in private prayer, in reading of the Scriptures, and in conversation with several most eminent clergymen of different denominations, from all whom she turned away in tearful disappointment, as they were too much engaged to enter into her case, to analize her feelings, to comprehend her difficulties and to have a personal and affectionate sympathy with her, in her darkness; still, in all these ways she strove, sorrowing; "faint, yet pursuing," to attain a more intimate acquaintance with a christian life; thus, when she came to die, every hope had a doubt; every aspiration was clouded with a fear; and yet, with these clogs, with her characteristic indomitable persistence, she did attain to a willingness to die; to one comprehensive desire, as she expressed it, "All I want now, is pardon and rest;—pardon and rest." And as if the spirit of the heavenly world was already enshrining her, she said, "I love everybody. I go in peace with God and man, with everybody." And these were among her last intelligible expressions.

### MALIGNANT CANCER.

When the body of this lady was examined, it was apparent from the very first glance that the disease was malignant cancer of the liver; that it must have been in progress a number of years; that it was inevitably fatal from the first moment of attack; and that no mortal power could have retarded the progress of the malady, even if its nature had been demonstrated from the very beginning. The comfort which these considerations have given surviving friends, is absolutely immeasurable; because it almost always happens that the bitterness of the grief which attends the loss of loved ones, is intensified by the conjecture that if something else had been done; or if something which was done had been done sooner, or not at all; or if some other physician had been called in; or the one in attendance had been called sooner—a different result might have followed.

To escape these harrowing reflections—which in many cases haunt the heart for a life-time afterward, eating out its life and gladness and withering it up like a flower without water—it would be greatly better to overcome the prejudice—almost universal—of examination after death, and thus know for certain in very many cases, the groundlessness of those distressing thoughts; with the further advantage, as some forms of disease are hereditary, important practical information, even vital in its character, may be had as to other members of the family in their after sickness;—to say nothing of that other consideration, that a surgical examination makes it perfectly certain that the body is without life, and being 'buried alive' is impossible.

BURIED ALIVE.

Washington, and other great names in ages past, had a great horror of being buried alive, and multitudes now living, have a fear more or less lively, that such may be their mishap, such fears being fed every now and then by statements, almost always apocryphal, of persons coming to life when on the point of being buried; or of cases of disinterment where bodies have been found having changed position; or have indicated a struggle, by the disarrangement of the grave clothes. It is the belief of the most eminent medical writers, that being buried alive does not occur twice in a million of cases, and that changes of position or condition of the clothing are easily accounted for in the generation of gases in the progress of decomposition; but as any one case may be that case in the million, and as affection recoils at the use of the surgeon's knife, on the dear body, still loved, tho' dead, perhaps the best general rule is to defer interment until the black discoloration of the skin in several parts, demonstrates that decomposition is going on, which can not be the case if there is a single spark of life remaining.

A LESSON.

Another practical lesson is, that thoughts of great bodily suffering in the last hour ought to be discountenanced from sound physiological reasoning. When the blood ceases to circulate in any part, there is no feeling in that part; and where there is no feeling there can be no suffering; besides, as circulation ceases at the extremities, the blood accumulates in the brain and deadens it, as in apoplexy ; the lungs, it is true, still work but it is only the motive power that is in operation ; sensation is all extinct, in cases of ordinary sickness, even where persons have been fatally injured while in vigorous health, and torturing pains are endured—these disappear before the final hour; and testimonies have reached us from minds of great strength, that when persons have recovered from hanging, drowning, and several other forms of death, they have remembered back to the moment of dissolution and have given descriptions of extatic sensations, so that in reality the evidence is all on the other side; and that if the dying have sensation or feeling, it is of a physically pleasurable character.

ANOTHER LESSON.

Nine-tenths of the readers of this Journal are christian men and women, and as we must all pass through the portals of death, sooner or later, and will have the mind exercised on the great point of moral preperation—one in comparison to which all others sink into nothingness,—another point in this lady's history is very suggestive. The character she sustained among those who knew her, was not merely that of a consistent member of a christian church from girlhood, but that of a scrupulous conscientious christian woman. In any promise of personal aid she always made it a point to exceed in what she agreed to; and as to pecuniary engagements to others, nothing short of the last cent, and at the appointed time, would satisfy her; but if coming to her, she was indifferent about small balances; and the very slightest intimation of tenants as to their disappointments, and difficulties in getting along, was sufficient to cause her to instruct her agent "not to be hard with them, nor exacting." Although educated in great laxity of views in reference to the observance of the Sabbath-day she learned a higher estimate and so taught her children, including habitual prayer and the daily reading of the Bible, while the sum of all business instructions was comprehended in less than half a dozen words to her children: "Be honorable; don't be mean;" and anything like deception, or taking advantage of another, always excited her strongest disapprobation; and yet, with all these traits, and with such a record, not a word or insinuation the most remote, ever escaped her, as expressive of any merit in her character; and when various persons at different times were inclined to encourage her to take a more favorable view of herself, she would invariably express herself afterwards, as to the unsatisfactory nature of such views. It never seemed to enter her imagination to claim anything in virtue of a blameless life; on the contrary, she felt abidingly, that she was no claiment; had no grounds for any hold on the notice and mercy of the Infinite One; and when the most beautiful "promises" were read to her she would listen with great interest, but then generally turn away, in tones so desponding as to draw tears from the eyes of others, men and women too: "But these are for the children of God: they are not for me, unworthy." At other times she would say, "That is to those who love God; I want to, I try to, but I fear that I do not, and that he don't love me. I am unworthy."

Very many comfort themselves as they approach the grave in the reflection, "I have no fear of death, or what is beyond the grave. God is just. I have tried to do my duty. I have never injured anyone, at least intentionly." And although they did not recognize the christian religion, or the obligations of the public ordinances; or of the Sabbath-day; nor regard themselves, in life, "on the Lord's side," they seem to have no regrets for shortcomings; for wrongs committed, for duties undone; while she, with a far different record, exclaimed with deepest feeling, "I have been worldly-minded, ambitious, and full of uncharitableness."

Whether it is better, in such solemn circumstances, to feel as Paul did, Ephesians 38, "less than the least of all saints," if a saint at all, or like the Pharisee, "I thank thee that I am not as other men are;" and whose chances are the better for attaining life immortal, let the reader judge. As for me, I would rather exclaim in Death, "God be merciful to me a sinner, for Jesus' sake."

FOR PHYSICIANS.

To the medical readers of the JOURNAL it will be interesting to know, that while the liver, when a man is in an erect position, reaches to the bottom of the lower ribs on the right side, its smaller division had been so pushed downwards and forwards by the cancerous deposit, that it was found in the centre of the abdomen, below the navel, and immediately under the skin. It was not eaten away, but seemed to be infiltrated by the cancerous growth to double its weight and size. The dejections for months were in form, but white; the skin became jaundiced; abdominal dropsy set in, and the official, immediate cause of death was "inanition;" emaciation and debility being the all-controlling features.

Such is a true record of HANNAH MATLACK. It is full of in struction and is exceedingly suggestive to a large class of minds. It will be read with absorbing and tearful interest by very many friends, scattered over our whole land, for she was a woman who so made a mark with those who came in contact with her that it is impossible that she should not be remembered with admiring interest.

